# Exercise Is Associated with Skeletal Muscle Autophagy–Lysosome-Related Markers and Nrf2/ARF1 Signaling in HFD Mice

**DOI:** 10.3390/nu18203329

**Published:** 2026-10-11

**Authors:** Shujuan Hu, Xuan Liu, Ruohan Xia, Xianwang Wang

**Affiliations:** 1 School of Education and Physical Education, Yangtze University, Jingzhou 434023, China; 500852@yangtzeu.edu.cn (S.H.);; 2 Department of Biochemistry and Molecular Biology, Center for Molecular Medicine, Health Science Center, Yangtze University, Jingzhou 434023, China

**Keywords:** exercise, muscle damage, Autophagy–Lysosome-Related Markers, Nrf2 signaling

## Abstract

**Purpose:** This study aimed to investigate the protective effects and potential mechanisms of exercise against long-term high-fat diet (HFD)-induced skeletal muscle dysfunction, with a focus on ADP-ribosylation factor 1 (ARF1)-related autophagy–lysosome markers and nuclear factor erythroid 2-related factor 2 (Nrf2) signaling. **Methods:** Forty male C57BL/6 mice were randomly divided into a normal diet group (DC) and an HFD group, and the latter was further subdivided into a model group (MG), exercise group (EG), Nrf2 inhibitor group (NG), and exercise combined with Nrf2 inhibitor group (ENG) after 12 weeks of HFD feeding. EG received 6 weeks of moderate-intensity treadmill exercise, NG was treated with Nrf2 inhibitor for 6 weeks, and ENG received both the 6-week treadmill exercise and inhibitor injections simultaneously. Serum lipid, blood glucose and insulin resistance indices were detected; skeletal muscle morphology was observed by H&E staining; mRNA and protein expression of ARF1, ULK1, ATP6V0A1 and other factors were determined by RT-qPCR, Western blotting and immunohistochemistry; the oxidative stress index was measured by an ELISA; and the colocalization of ARF1/ATP6V0A1 with the lysosomal marker Lamp1 was analyzed by immunofluorescence. **Results:** Long-term HFD significantly increased mouse body weight, serum TC, TG, LDL-C, FBG levels and the HOMA-IR index, induced morphological alterations in skeletal muscle (loose myofiber arrangement, abundant intracellular vacuoles), inhibited the mRNA expression of ARF1, ULK1 and ATP6V0A1, and elevated malondialdehyde (MDA) content (*p* < 0.05 or *p* < 0.01). Six weeks of moderate-intensity treadmill exercise effectively improved HFD-induced metabolic disorders and skeletal muscle morphological damage, upregulated the expression of ARF1, ULK1 and ATP6V0A1, and reduced oxidative stress (*p* < 0.05 or *p* < 0.01). Notably, luteolin-mediated inhibition of Nrf2 signaling may partially reverse exercise-induced alterations in ARF1-related autophagy–lysosome markers and skeletal muscle morphology. **Conclusions:** Long-term HFD may induce morphological alterations in skeletal muscle, accompanied by changes in ARF1-associated autophagy–lysosome markers and elevated oxidative stress. Conversely, moderate-intensity exercise may ameliorate HFD-related changes in skeletal muscle morphology, potentially via Nrf2 signaling, providing a potential molecular target and theoretical basis for the exercise intervention strategies against HFD-induced metabolic-related skeletal muscle dysfunction.

## 1. Introduction

Excessive intake of high-fat diets (HFDs) serves as a major driving factor for the growing prevalence of obesity, metabolic disorders, cardiovascular diseases, and various malignant tumors. When skeletal muscle suffers from HFD-stimulated metabolic disturbance, oxidative stress and inflammatory status tend to become aggravated, which further promotes muscle-mass loss [1]. Aerobic exercise can mitigate HFD-induced skeletal muscle damage by enhancing antioxidant capacity and mitochondrial protective function [2]. Autophagy is an essential catabolic process responsible for intracellular organelle quality control and metabolic homeostasis, and its dysfunction is closely associated with skeletal muscle metabolic disorders [3]. Autophagic lysosome reformation (ALR), a critical downstream procedure of autophagy, sustains continuous autophagic flux by regenerating functional lysosomes from autolysosome membranes, which is indispensable for maintaining normal skeletal muscle autophagic activity and cellular homeostasis [4].

ADP-ribosylation factor 1 (ARF1), a small Ras-family GTPase, serves as a key molecular switch that governs intracellular membrane trafficking and lipid metabolism. It regulates multiple biological processes, including intracellular protein and lipid sorting and trafficking, and modulates lipolysis pathways to maintain cellular metabolic homeostasis [5,6]. ARF1 further controls fatty acid storage and utilization, mediates inter-organelle communication, and thereby sustains fatty acid metabolism and mitochondrial energy homeostasis [7,8]. Notably, ARF1 participates in lysosomal membrane fusion, and elevates the activity of mammalian target of rapamycin (mTOR) on autophagosomes, consequently modulating lysosomal degradation efficiency and lipid metabolism [9]. Following autophagy initiation, mTOR reaccumulates on autophagolysosomes and promotes ARF1 recruitment. In turn, ARF1 activity modulates the reactivation of mTOR, which governs autophagy termination and lysosome biogenesis. Collectively, ARF1 facilitates autophagolysosome recycling and maintains stable autophagic flux [10]. Moreover, lysine-73 undergoes lactylation modification (ARF1-Kla73), enhances mitochondrial trafficking and exerts protective effects against cellular damage, indicating a vital post-translational modification for metabolic adaptation [11].

Nuclear factor (erythroid-derived 2)-related factor 2 (Nrf2) serves as a master antioxidant transcription factor that defends cells against oxidative and inflammatory damage. Under stress conditions, Nrf2 activates the transcription of downstream antioxidant and anti-inflammatory genes, thereby improving cellular metabolic adaption [12]. Multiple experimental studies have demonstrated that physical exercise alleviates chronic-inflammation-related metabolic complications in patients with obesity and non-alcoholic fatty-liver disease (NAFLD). By attenuating inflammation-induced insulin resistance and type 2 diabetes, physical activity exerts preventive effects and improves therapeutic outcomes [13,14,15]. Chronic resistance training combined with nutritional timing can enhance skeletal muscle mass and strength by inhibiting PTEN and activating the PI3K-AKT-mTOR and FoxO signaling pathways [16]. Resistance training may attenuate skeletal muscle atrophy in a mouse model of type 2 diabetes mellitus (T2DM), potentially via upregulation of fibroblast growth factor 21 (FGF21) and subsequent activation of the PI3K/Akt pathway [17]. Lactic acid produced during exercise undergoes lactylation modification of the mTOR protein in cells. The modified mTOR further activates skeletal muscle autophagy, which improves muscle adaptation to physical training and preserves metabolic wellbeing [18]. Taken together, this evidence confirms that exercise exerts protective effects against HFD-induced skeletal muscle metabolic damage via modulating oxidative stress and autophagy. However, whether Nrf2-mediated antioxidant signaling interacts with the alterations in ARF1- and autophagy-related markers to mediate exercise-induced skeletal muscle protection under HFD stress remains uncharacterized.

This work investigates ARF1 changes under HFD-induced oxidative stress, a condition that brings about muscle-mass loss, impaired insulin sensitivity and weakened exercise capacity, with defective autophagy acting as one central contributing mechanism. Our aim is to explore associations between exercise and HFD-related molecular and morphological changes in skeletal muscle, together with alterations in ARF1- and autophagy-related markers. Our hypothesis is that exercise is linked to changes in Nrf2- and ARF1-associated autophagy–lysosome markers in HFD-fed mice, and that luteolin may modify these exercise-related molecular correlates by modulating Nrf2 signaling. Our observations offer molecular clues for understanding exercise-associated skeletal muscle adaptations under HFD conditions.

## 2. Materials and Methods

### 2.1. Experimental Animals

This study utilized 40 male C57BL/6 mice, aged 7 weeks, which were purchased from the Animal Research Center of China Three Gorges University and housed at the Animal Center of Yangtze University (Jingzhou, China). All mice were randomly assigned to either a normal diet group (DC group, *n* = 8) or a high-fat diet group (HFD group, *n* = 32) using a simple random sampling method. The DC group was consistently fed with regular feed, while the HFD group was fed with high-fat feed (12492M, 60% fat content, purified type, Beijing Boai Gang Biotechnology Co., Ltd., Beijing, China). After 12 weeks, the mice in the HFD group were further randomly subdivided into four groups (*n* = 8 per group): a model group (MG group), an exercise group (EG group), an Nrf2 inhibitor group (NG group), and an exercise plus Nrf2 inhibitor group (ENG group). During the animal treatment, feeding, and sample collection procedures, the researchers were blinded to the group information. All experimental protocols were approved by the Animal Experimental Ethics Committee of Yangtze University (approval no. 202401025, date: 10 July 2024).

### 2.2. Treadmill Exercise Training and Sample Collection

The exercise intervention protocol [19,20] and intraperitoneal luteolin administration protocol [21] were modified from previously reported methods. The exercise group underwent moderate-intensity treadmill running. The specific protocol was as follows: (1) An adaptive treadmill running period lasting 5 days: the exercise intensity was set at 10 m/min for the first 3 days, and then adjusted to 12 m/min and 14 m/min on the following two days, respectively. The daily exercise duration was 30 min. (2) Following the adaptation period, the mice underwent a 6-week moderate-intensity treadmill running program at a 0° incline, with the intensity maintained at 12 m/min for 60 min per day, 5 days per week (Saturday and Sunday off). The Nrf2 inhibitor intervention protocol was as follows: The inhibitor (luteolin, HY-N0162, MedChemExpress, Monmouth Junction, USA) was dissolved in 10% DMSO + 90% 20% sulfobutylether-β-cyclodextrin (SBE-β-CD) in saline. Luteolin was administered at a dose of 20 mg/kg, with an injection volume of 10 mL/kg. It was administered by intraperitoneal injection, with the injection regimen lasting for 6 weeks, with 5 injections per week. The vehicle group received an equal volume of the identical solvent mixture (10% DMSO + 90% 20% SBE-β-CD in saline) following an identical injection schedule, volume and frequency, matching the luteolin-treated groups.

At 24 h after the last exercise session and after a 12 h overnight fast, mice were anesthetized by inhalation of isoflurane for 5 min. Blood samples were collected from the retro-orbital venous plexus under deep-anesthetic conditions. Subsequently, mice were fully euthanized via intraperitoneal injection of Avertin (20 uL/g, Dowobio, Shanghai, China, DW3101). After intraperitoneal injection of Avertin, mice were monitored until complete loss of the pedal withdrawal reflex and corneal reflex as well as cessation of spontaneous breathing to confirm successful euthanasia. Blood samples were allowed to stand at room temperature for 30 min, then centrifuged at 3000 rpm for 15 min to obtain the serum supernatant for subsequent analysis. The quadriceps femoris muscles were rapidly excised; one portion was placed in fixative solution (the main components are formalin, acetic acid and anhydrous ethanol, P0003, Pinuofei Biotechnology, Wuhan, China) for morphological examination, and the other portion was immediately flash-frozen in liquid nitrogen before being transferred to a −80 °C freezer for storage until further analysis.

### 2.3. Biochemical Indicator Analysis

Total cholesterol (TC), triglycerides (TGs), low-density lipoprotein cholesterol (LDL-C), and high-density lipoprotein cholesterol (HDL-C) were detected using a microplate assay kit (Biyun Tian Biotechnology Co., Ltd, Shanghai, China). An ELISA kit was used to detect the contents of fasting insulin (FINS) in the serum of each group of mice (Xiamen Lunchangshuo Biotechnology Co., Ltd., Xiamen, China). Homeostatic Model Assessment of Insulin Resistance (HOMA-IR) = FINS (μU/mL) × FBG (mmol/L)/22.5.

### 2.4. Hematoxylin and Eosin (H&E) Staining

Muscle tissues were fixed in muscle fixative solution for 48 h and rinsed under running water for 6 h. After dehydration in a graded ethanol series, clearing in xylene, and embedding in paraffin, 4 μm sections were prepared and baked at 60 °C for 30 min. Following routine deparaffinization and rehydration, the sections were stained with hematoxylin and eosin (H&E). After dehydration, clearing, and mounting with neutral balsam, sections were observed and photographed using an Olympus BX51 optical microscope (Olympus, Tokyo, Japan). For histological assessment, over five non-overlapping random high-magnification fields were imaged per section using an optical microscope at a consistent magnification. Biological replicates were included to guarantee the reliability of pathological analysis. The proportion of the muscle-tissue area was quantified using ImageJ software (ImageJ version 1.54f).

### 2.5. Fluorescence Immunohistochemistry Staining

Muscle-tissue sections were dewaxed in xylene and anhydrous ethanol and then rinsed with water. Antigen retrieval was performed in pre-heated 1× citric-acid buffer (pH 6.0) in a pressure cooker for 2 min, followed by cooling to room temperature. The sections were blocked with 10% matched goat serum at 37 °C for 30 min. After overnight incubation with diluted primary antibodies at 4 °C, corresponding secondary antibodies (1:100 in PBST) were incubated at 37 °C for 1 h. After DAPI staining and repeated PBS washing, sections were mounted and stored at 4 °C in darkness. Images were acquired using a Nikon ECLIPSE Ci microscope (Nikon, Tokyo, Japan), and fluorescence intensity was analyzed with ImageJ software. There were 3 non-consecutive sections per animal, and 2–3 non-overlapping fields per section.

### 2.6. Real-Time Fluorescence Quantitative PCR Detection

Total RNA was extracted from 100 mg of mouse muscle tissue using TRIzol (R411-01, Vazyme, Nanjing, China). Reverse transcription was performed to synthesize cDNA using a commercial kit (RR047A, TAKARA, Shiga, Japan). Quantitative real-time PCR (qRT-PCR) was conducted on an ABI 7500 system (ThermoFisher Scientific, Waltham, MA, USA) with a SYBR Green kit (RR420A, TAKARA). Specific primer sequences for the target and reference genes are provided in Table 1.

### 2.7. Western Blotting

Quadriceps femoris muscle samples (0.1 g) were collected for protein extraction. The protein samples were separated by 10% SDS-PAGE and subsequently transferred onto a PVDF membrane using the wet transfer method. The membrane was blocked with 5% skimmed milk for 1 h at room temperature, followed by overnight incubation at 4 °C with the corresponding primary antibody (diluted 1:1000). The primary antibodies used were as follows: ARF1 antibody (YP-mAb-18323, Upingbio, Hangzhou, China), ATP6V0A1 antibody (YP-mAb-18049, Upingbio), LDH-A antibody (R24822, Zenbio, Chengdu, China), LDH-B antibody (R389134, Zenbio), ULK1 antibody (R381887, Zenbio), and β-actin antibody (4970S, Cell Signaling, Danvers, MA, USA). After washing, the membrane was incubated with HRP-conjugated goat anti-rabbit IgG (A0208, Beyotime Biotechnology, Shanghai, China) secondary antibody (diluted 1:10,000) for 2 h at room temperature on a shaker. Protein bands were visualized using a chemiluminescence imaging system (ChemiDoc MP System, Bio-Rad Laboratories, Inc., Hercules, CA, USA).

### 2.8. Statistical Analysis

All experimental data were analyzed using SPSS software 26.0. Data are presented as the mean ± standard deviation (M ± SD). Comparisons among multiple groups were performed using one-way analysis of variance (ANOVA), with Tukey HSD post hoc comparisons. Weekly body weight was analyzed using the repeated measurement method. Sample size was selected based on published work and lab experience. Western blotting results were quantified using ImageJ software. (Briefly, background signals were subtracted initially. The relative protein expression was determined as the ratio of the grayscale intensity of the target protein to β-actin within each identical sample). The histograms were generated using GraphPad Prism 8.0 software.

## 3. Results

### 3.1. A Long-Term High-Fat Diet Can Induce a Series of Damages to Skeletal Muscles

**Figure 1:**
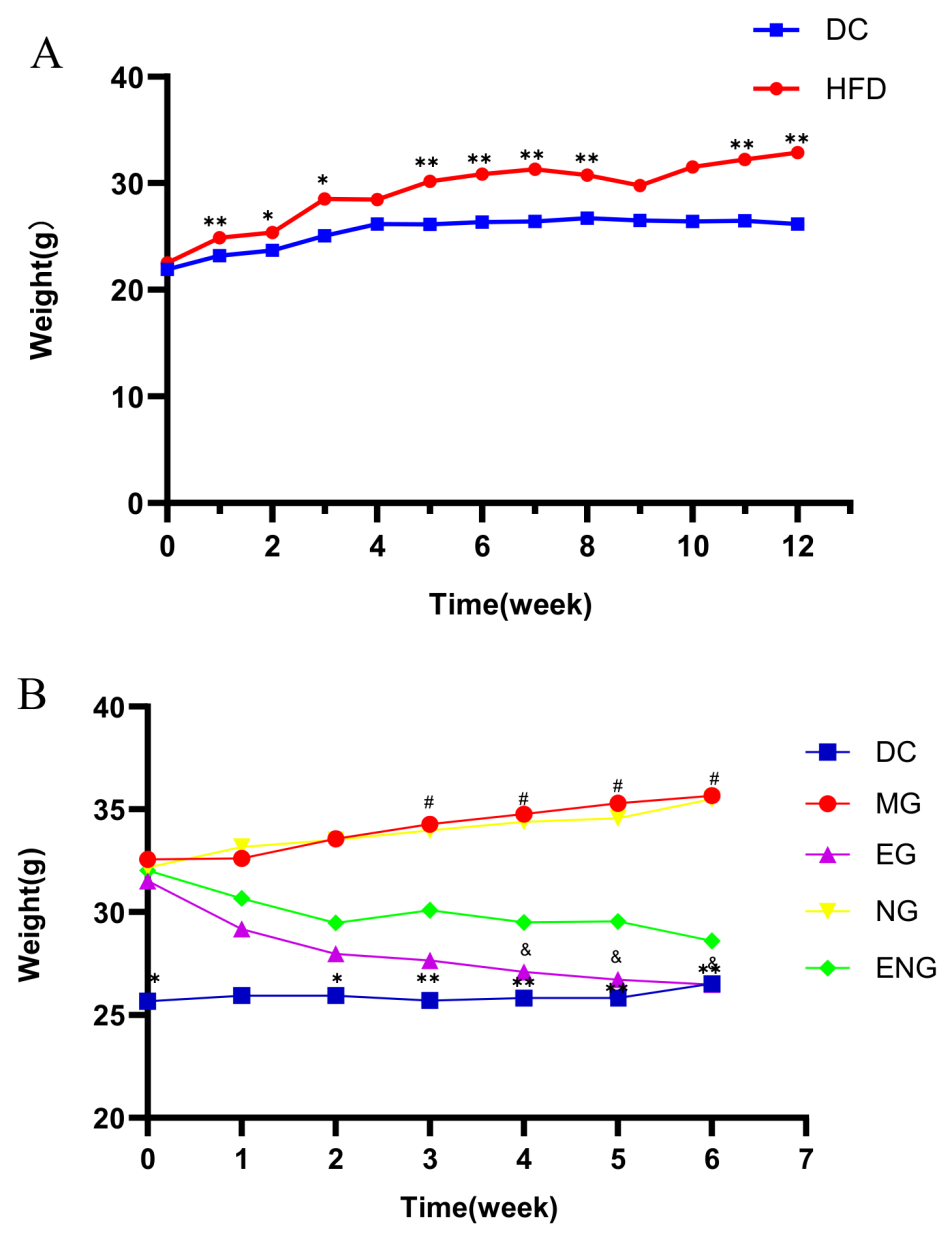
**Changes in the body weight of each group of mice:** (**A**) the weight changes of the DC and HFD groups before the intervention, * *p* < 0.05, ** *p* < 0.01 vs. DC; (**B**) the changes in body weight of different groups after the intervention, * *p* < 0.05, ** *p* < 0.01 vs. MG; ^#^ *p* < 0.05 vs. EG; ^&^ *p* < 0.05 vs. NG. DC, normal diet group; HFD, high-fat diet group; MG, model group; EG, exercise group; NG, Nrf2 inhibitor group; ENG, exercise plus Nrf2 inhibitor group.

**Figure 2:**
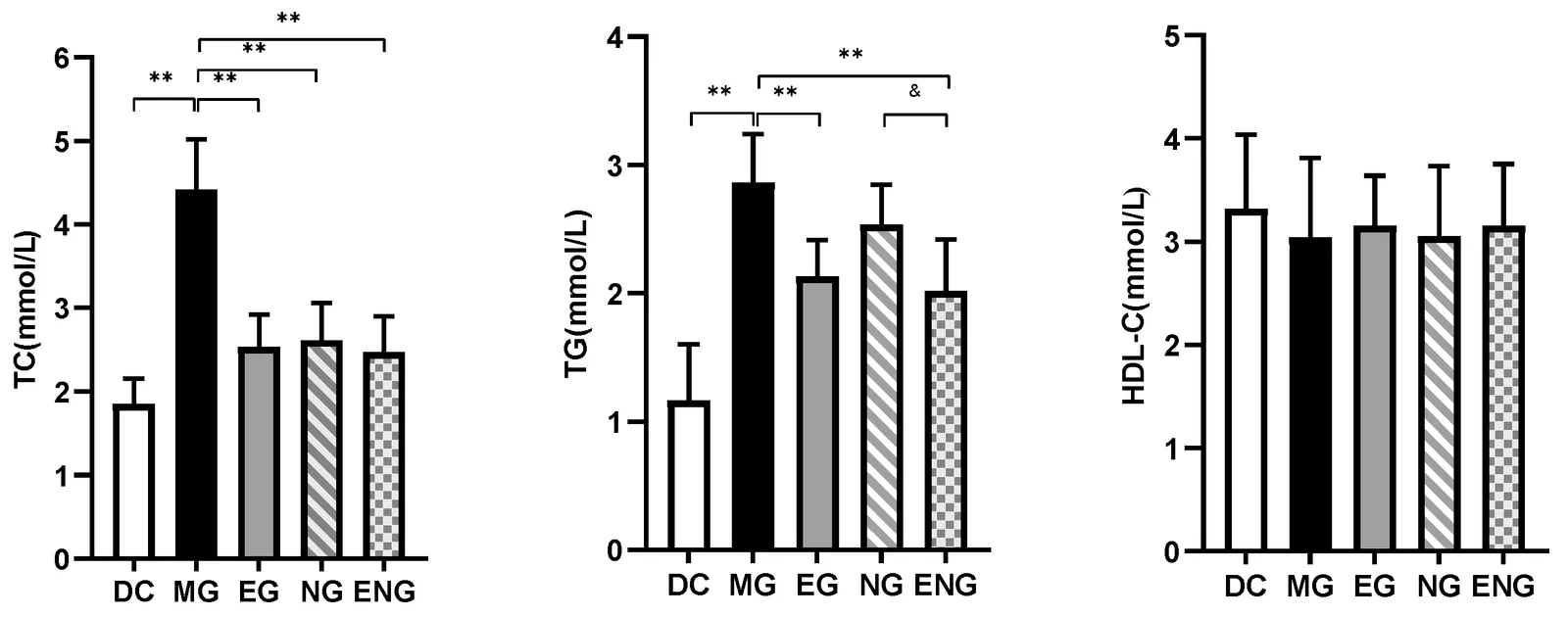

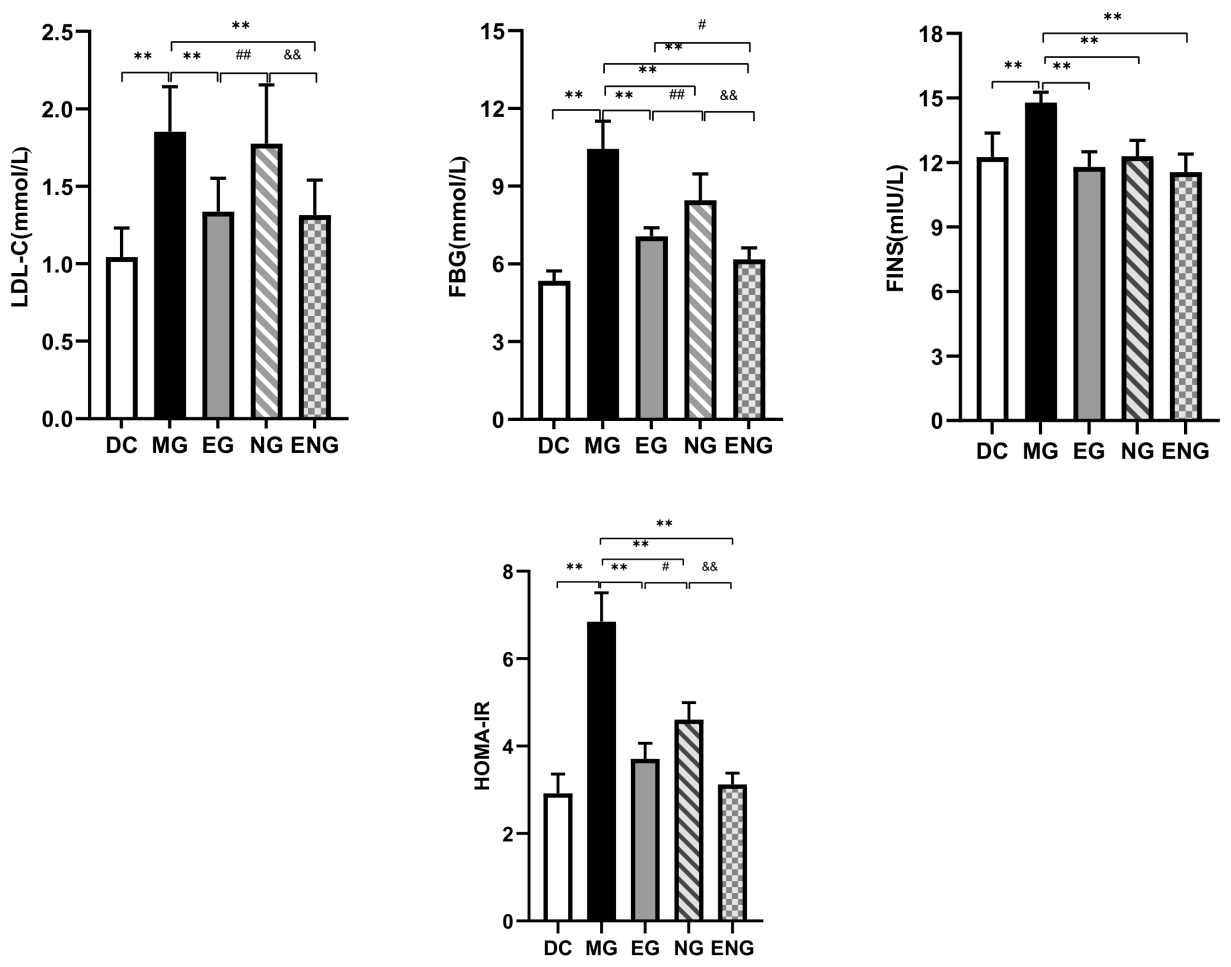
**Changes in blood glucose, blood lipids and IR indicators in HFD mice after intervention. *n* = 8 mice per group.** ** *p* < 0.01 vs. MG; ^#^ *p* < 0.05, ^##^ *p* < 0.01 vs. EG; ^&^ *p* < 0.05, ^&&^ *p* < 0.01 vs. NG. DC, normal diet group; HFD, high-fat diet group; MG, model group; EG, exercise group; NG, Nrf2 inhibitor group; ENG, exercise plus Nrf2 inhibitor group.

**Figure 3:**
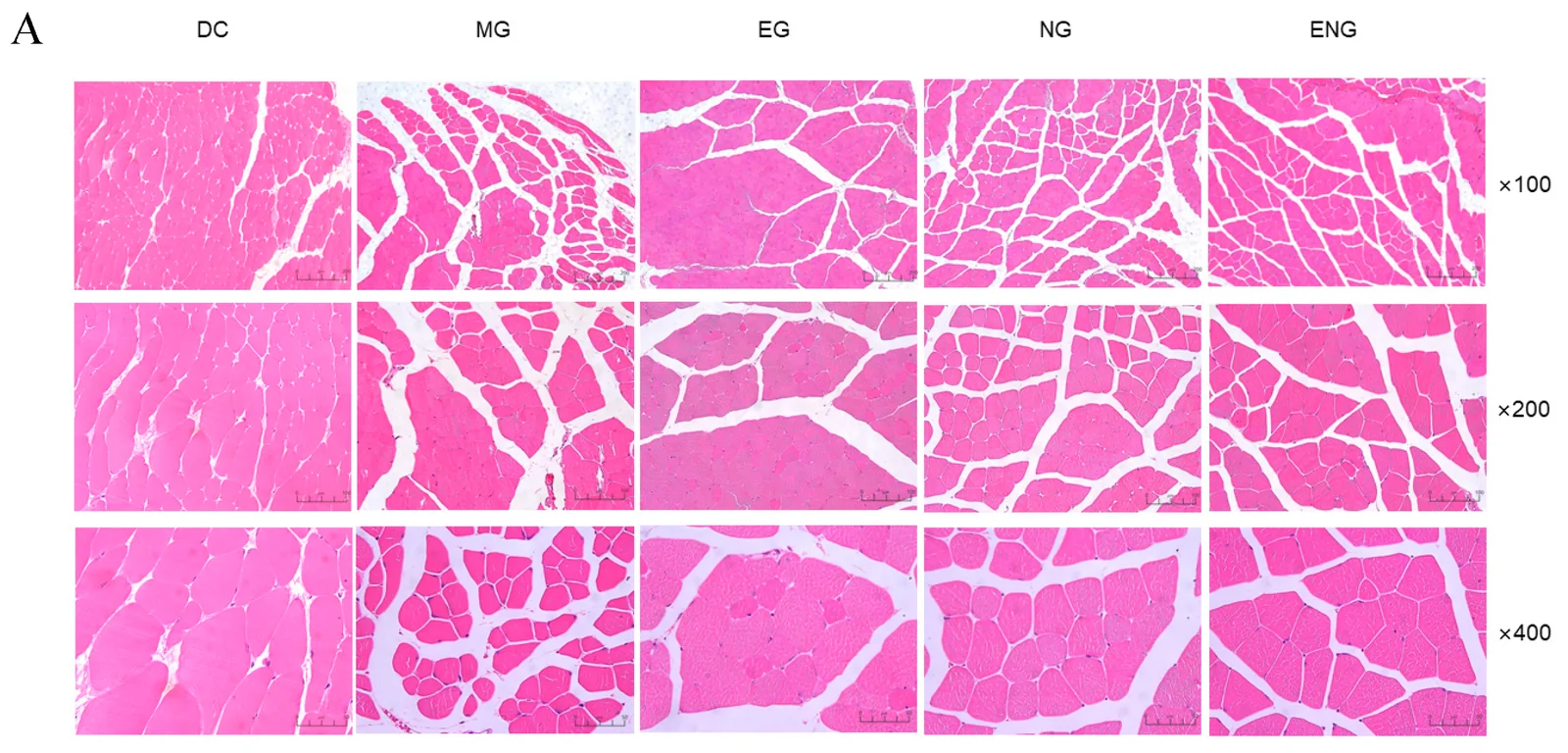

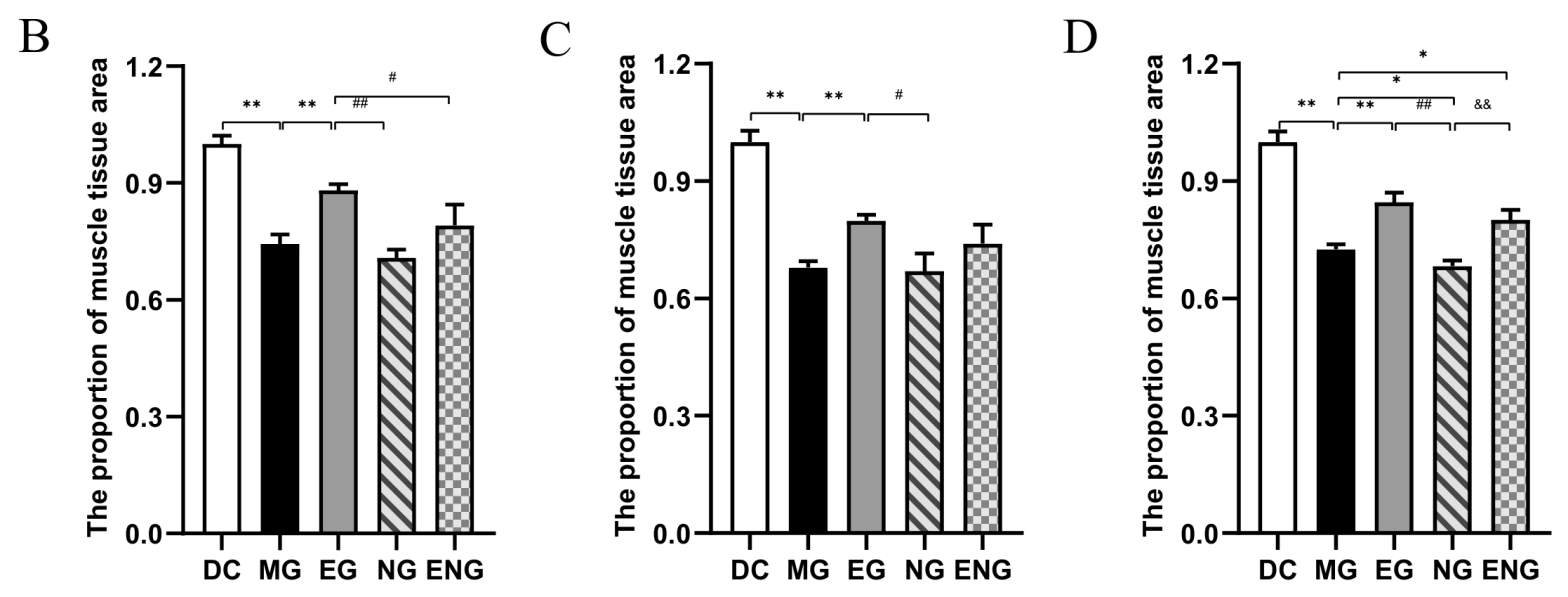
**Morphological changes in the skeletal muscle of HFD mice after intervention. *n* = 3 mice per group.** (**A**). H&E staining of skeletal muscle. The scale bars are 200 μm, 100 μm and 50 μm for 100×, 200× and 400× magnifications, respectively. (**B**–**D**). The proportion of the muscle-tissue area. (**B**–**D**) represent the proportions of the muscle-tissue area at 100×, 200×, and 400× magnifications, respectively. * *p* < 0.05, ** *p* < 0.01 vs. MG; ^#^ *p* < 0.05, ^##^ *p* < 0.01 vs. EG; ^&&^ *p* < 0.01 vs. NG. DC, normal diet group; HFD, high-fat diet group; MG, model group; EG, exercise group; NG, Nrf2 inhibitor group; ENG, exercise plus Nrf2 inhibitor group.

As shown in Figure 1A, after one week of adaptive feeding in both the DC group and the HFD group, the HFD group was fed a high-fat diet. During weeks 1–12, the body weight of the HFD groups was significantly higher than that of the DC group (*p* < 0.05 or *p* < 0.01), indicating a marked increase in body weight in mice fed with HFD. As shown in Figure 2, serum levels of TC, TG, LDL-C, FBG, FINS and HOMA-IR were significantly higher in the MG group than in the DC group (*p* < 0.01). H&E staining of skeletal muscle revealed that the DC group exhibited intact muscle cross-sections with well-preserved cell morphology. Muscle cells were separated by white connective tissue, with regular morphology and relatively tight arrangement. In contrast, the MG group displayed loosely arranged muscle cells with numerous intracellular vacuoles and irregular tissue morphology, accompanied by a significant reduction in the proportional area of muscle tissue (*p* < 0.01). These results indicate that long-term HFD induces molecular and morphological alterations in skeletal muscle (Figure 3).

### 3.2. HFD Correlates with Changes in ARF1-Associated Autophagy–Lysosome Markers in Skeletal Muscle

ARF1 is normally localized to the Golgi apparatus. Upon autophagy activation, ARF1 translocates from the Golgi to the autolysosomal membrane [10]. The activity of ARF1 affects the activation of mTOR, which serves as a signal to terminate autophagy and initiate ALR [10,22]. ULK1 (unc-51-like kinase 1) is a serine/threonine protein kinase that plays a critical role in the initiation stage of autophagy [23]. Activation of ULK1 induces autophagy [24]. ATP6V0A1 is an essential subunit of the intracellular V-ATPase proton pump, and is essential for regulating intracellular pH, maintaining the acidic environment of lysosomes, and participating in multiple cellular processes [25].

**Figure 4:**
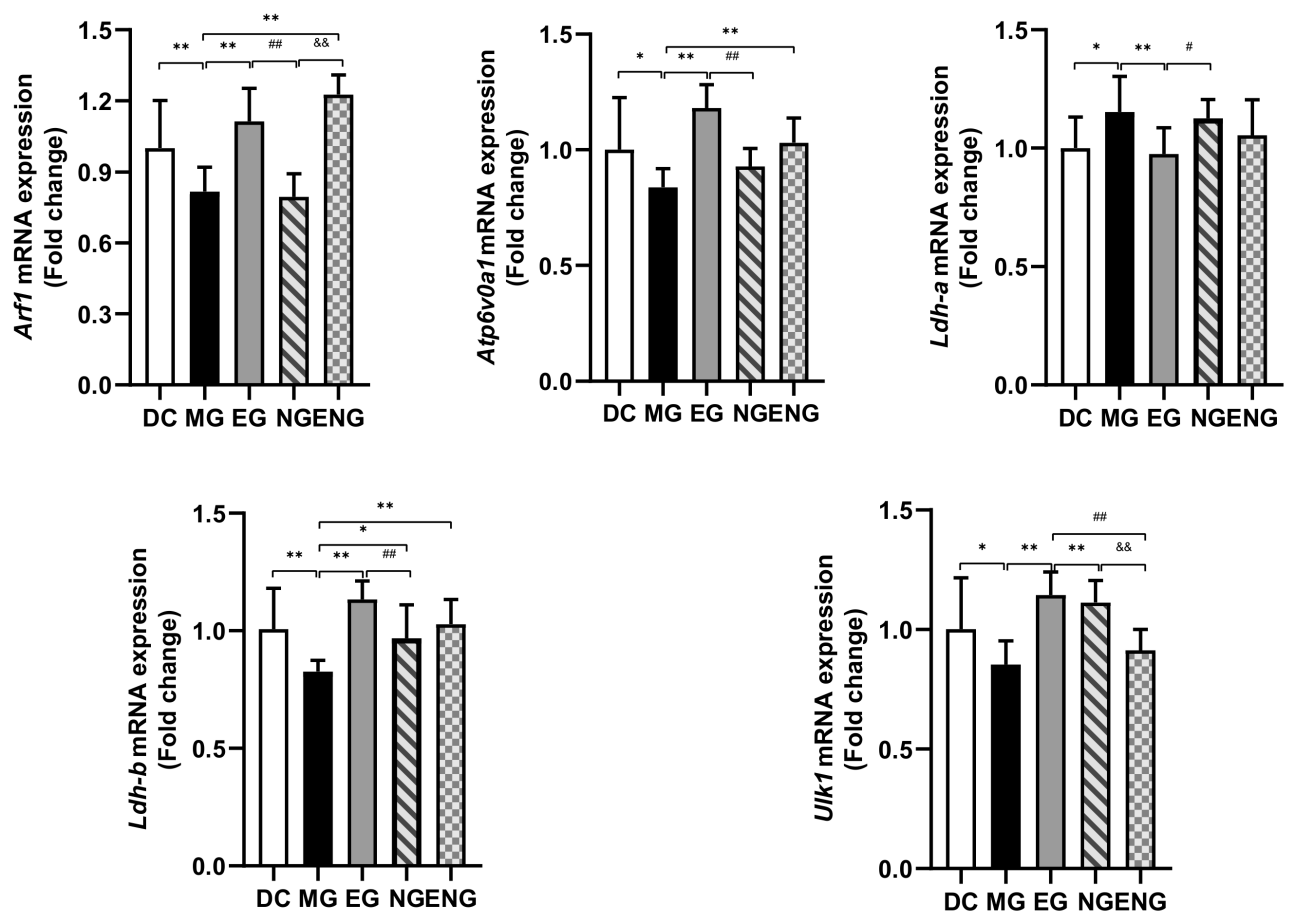
**Changes in the mRNA levels of autophagy-related factors in the skeletal muscle of HFD mice after intervention. *n * = 8 mice per group.** * *p* < 0.05, ** *p* < 0.01 vs. MG; ^#^ *p* < 0.05, ^##^ *p* < 0.01 vs. EG; ^&&^ *p* < 0.01 vs. NG. DC, normal diet group; HFD, high-fat diet group; MG, model group; EG, exercise group; NG, Nrf2 inhibitor group; ENG, exercise plus Nrf2 inhibitor group.

Long-term HFD causes sustained activation of mTORC1, thereby inhibiting autophagy initiation. Meanwhile, key steps in the late stage of autophagy, such as ALR, are blocked [26]. As shown in Figure 4, compared with the DC group, the MG group exhibited significantly decreased mRNA expression levels of *Arf1*, *Atp6v0a1*, *Ulk1*, and *Ldh-b* (*p* < 0.05 or *p* < 0.01), whereas mRNA expression of *Ldh-a* was markedly increased (*p* < 0.05 or *p* < 0.01).

**Figure 5:**
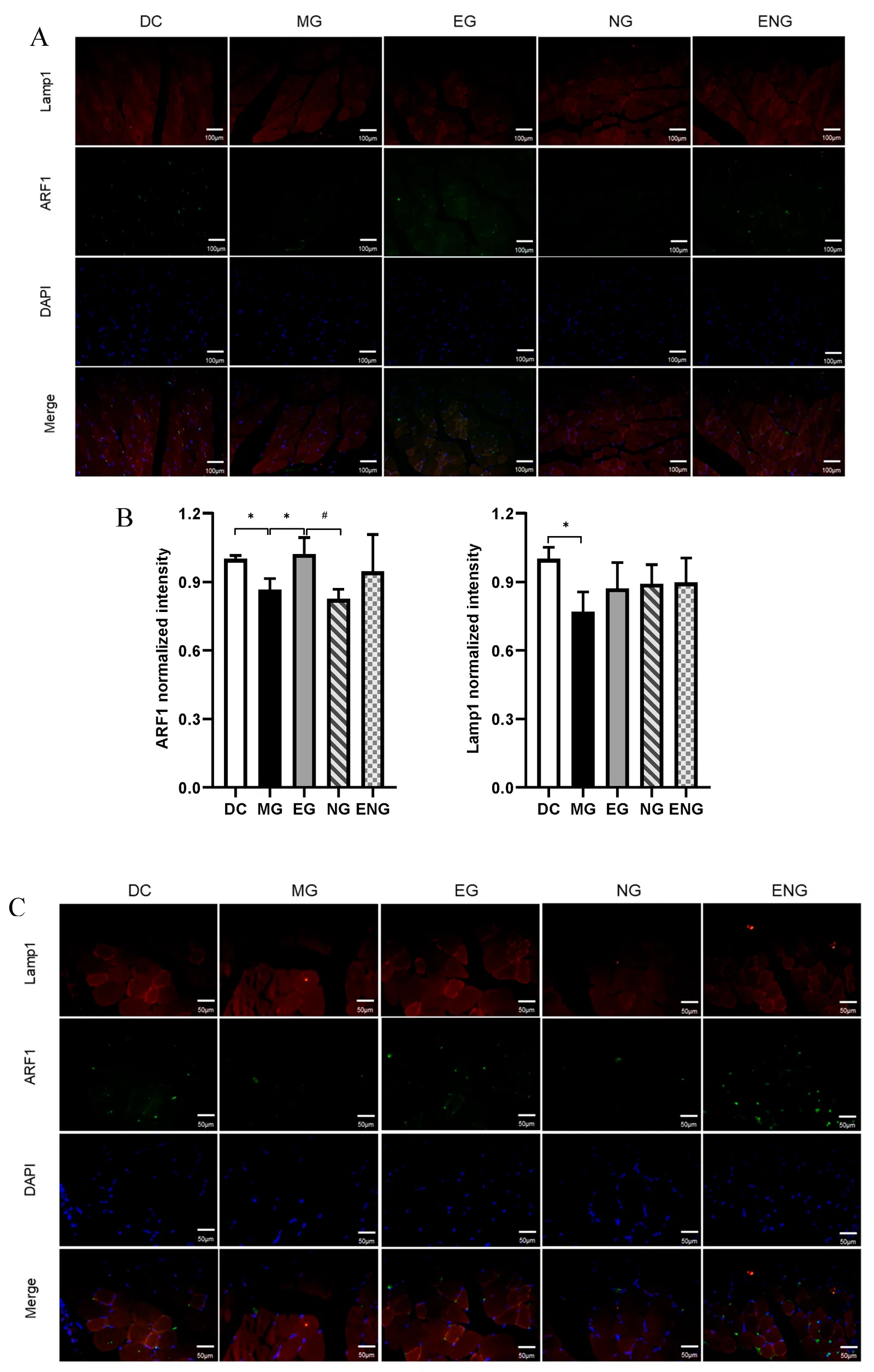

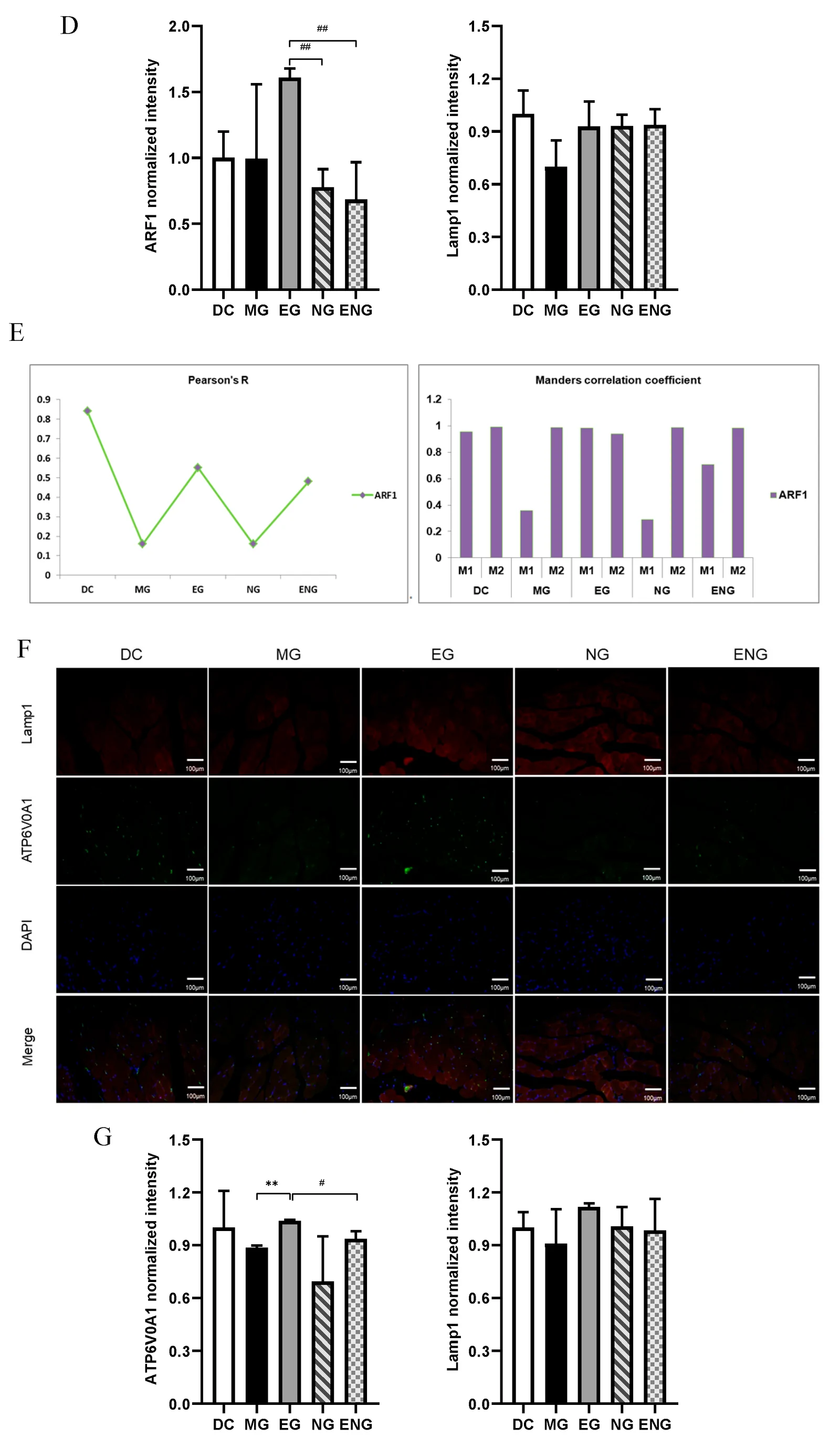

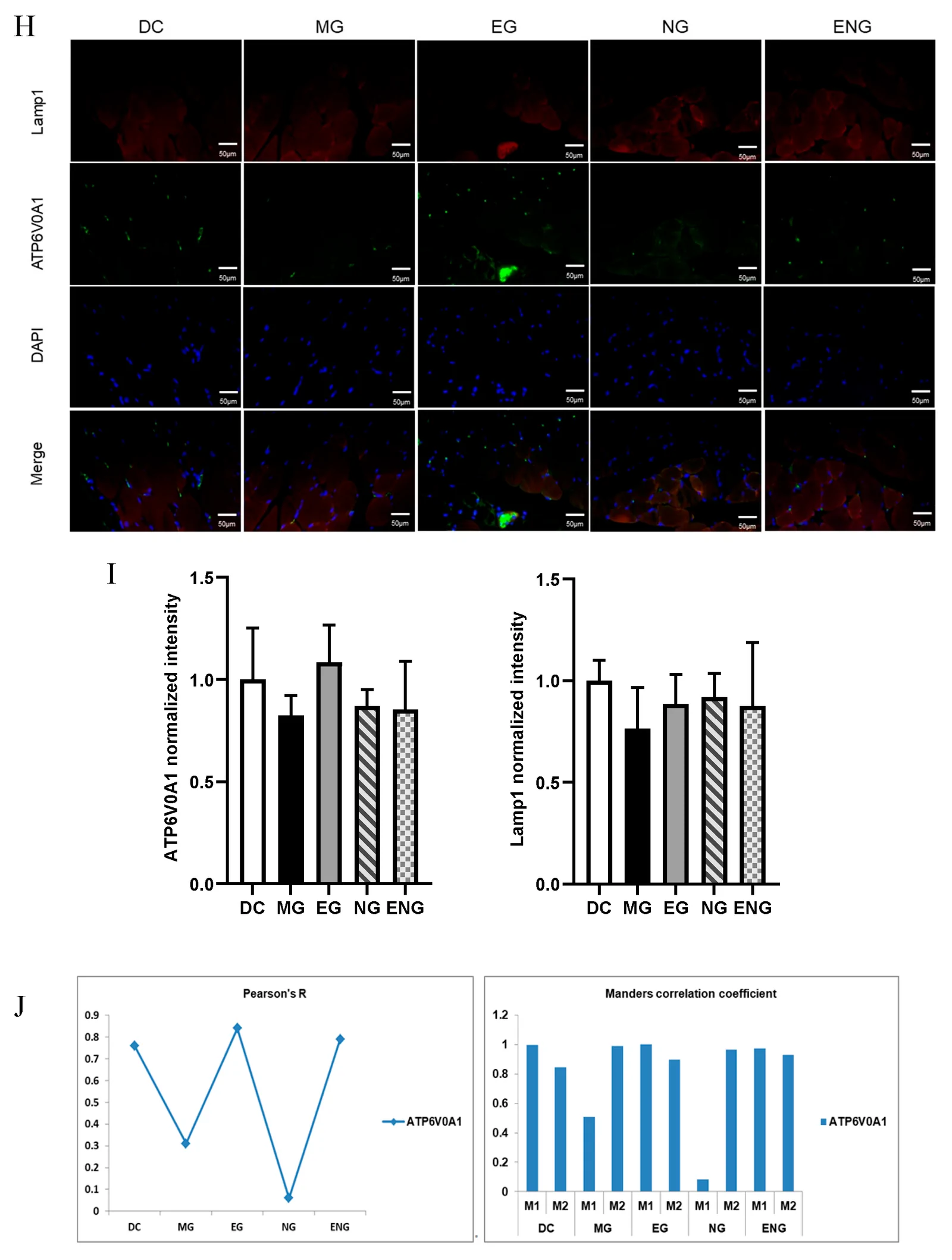
**Results of immunofluorescence staining of skeletal muscle in HFD mice after intervention. *n* = 3 mice per group.** (**A**). The expression of ARF1 and Lamp1 in skeletal muscle; the scale bars are 100 μm for 200× magnifications. (**B**). The normalized intensities of ARF1 and Lamp1 at 200 magnification. (**C**). The expression of ARF1 and Lamp1 in skeletal muscle; the scale bars are 50 μm for 400× magnifications. (**D**). The normalized intensities of ARF1 and Lamp1 at 400 magnification. (**E**). The Pearson correlation coefficient and Manders correlation coefficient between ARF1 and Lamp1. The closer the Pearson correlation coefficient value is to 1, the stronger the spatial correlation between Lamp1 and ARF1 (indicating a positive correlation). The Manders correlation coefficient represents the colocalization coefficient between ARF1 and Lamp1. M1 indicates the proportion of colocalization between ARF1 and Lamp1; M2 indicates the proportion of colocalization between Lamp1 and ARF1. If the ratio of M1/M2 is greater than 0.6, it indicates the presence of the colocalization phenomenon, while if the ratio of M1/M2 is less than 0.3, it indicates the absence of the colocalization phenomenon. (**F**). The expression of ATP6V0A1 and Lamp1 in skeletal muscle; the scale bars are 100 μm for 200× magnifications. (**G**). The normalized intensities of ATP6V0A1 and Lamp1 at 200 magnification. (**H**). The expression of ATP6V0A1 and Lamp1 in skeletal muscle; the scale bars are 50 μm for 400× magnifications. (**I**). The normalized intensities of ATP6V0A1 and Lamp1 at 400 magnification. (**J**). The Pearson correlation coefficient and Manders correlation coefficient between ATP6V0A1 and Lamp1. * *p* < 0.05, ** *p* < 0.01 vs. MG; ^#^ *p* < 0.05, ^##^ *p* < 0.01 vs. EG. DC, normal diet group; HFD, high-fat diet group; MG, model group; EG, exercise group; NG, Nrf2 inhibitor group; ENG, exercise plus Nrf2 inhibitor group.

Lamp1 (lysosome-associated membrane protein 1) is a membrane glycoprotein mainly localized to the lysosomal membrane. The observed colocalization of ARF1 and Lamp1 suggests an association between ARF1 and autophagic lysosomes, which may be relevant to ALR [27]. We performed double immunofluorescence staining using the lysosomal marker Lamp1 together with ARF1 or ATP6V0A1. The results showed that, compared with the DC group, the expressions of ARF1 and ATP6V0A1 in the lysosomes of skeletal muscle were lower, and their colocalization was weaker in the MG group (Figure 5E,J).

**Figure 6:**
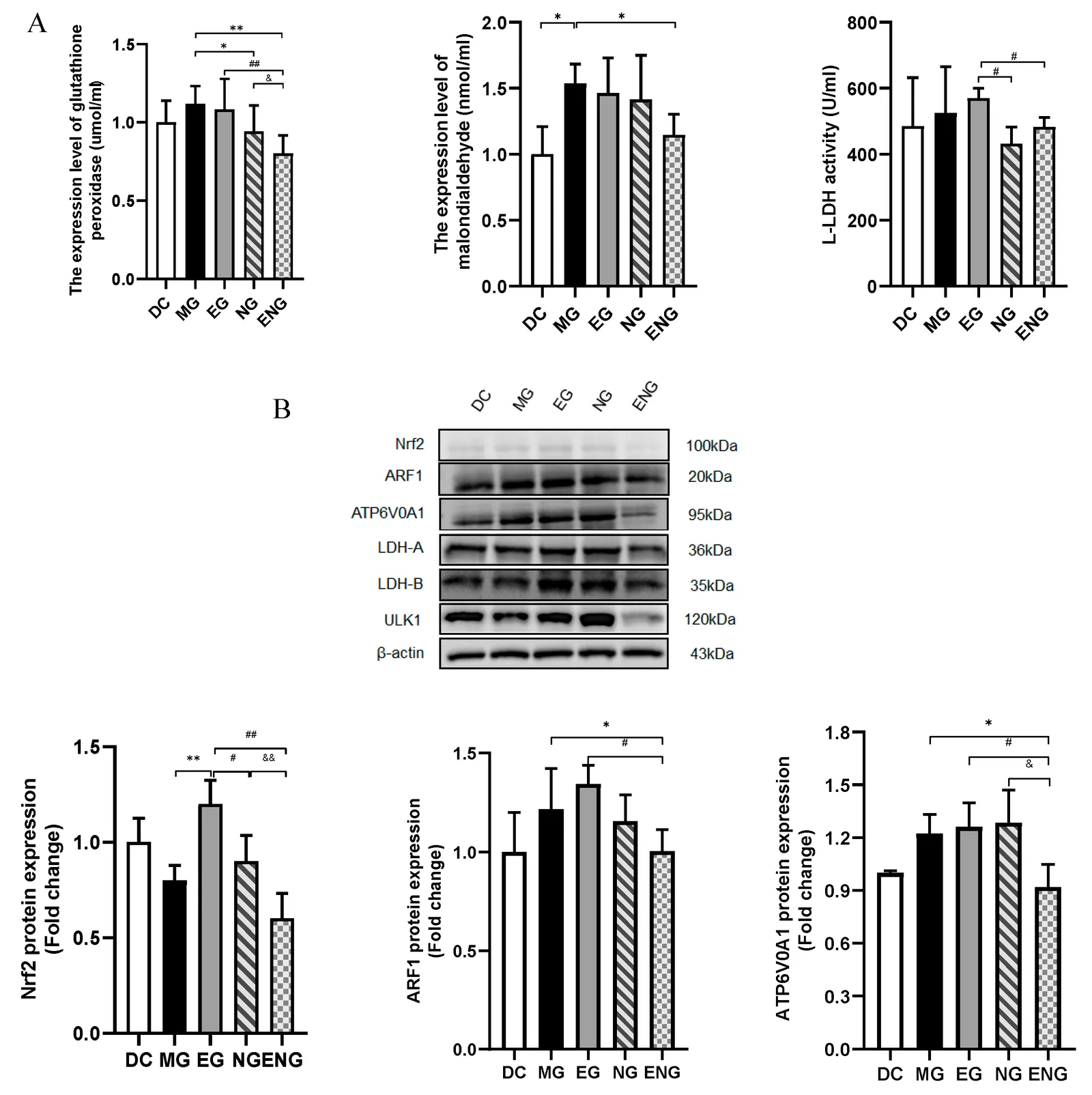

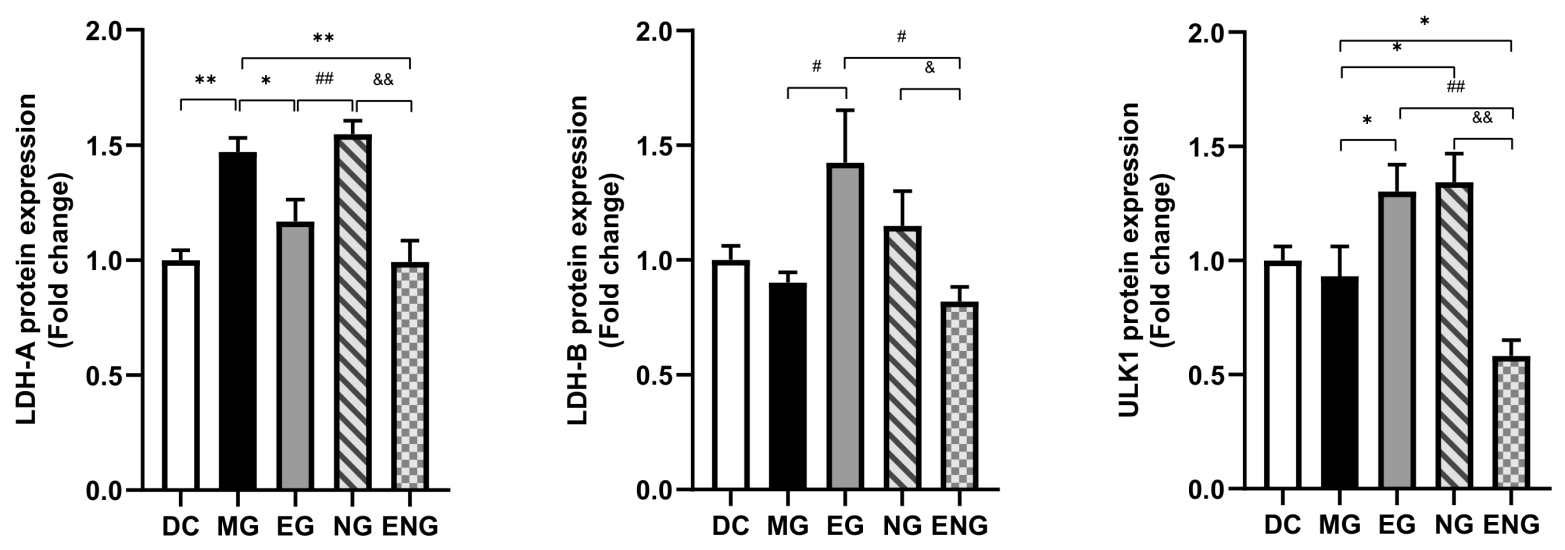
**Changes in the protein expression levels of autophagy-related factors in the skeletal muscles of HFD mice after intervention. *n* = 3 mice per group.** (**A**). ELISA analysis was performed to detect the expression levels of MDA and other factors. (**B**). Western blotting analysis was performed to detect the protein expression levels of ARF1 and related factors in the skeletal muscle of mice. * *p* < 0.05, ** *p* < 0.01 vs. MG; ^#^ *p* < 0.05, ^##^ *p* < 0.01 vs. EG; ^&^ *p* < 0.05, ^&&^ *p* < 0.01 vs. NG. DC, normal diet group; HFD, high-fat diet group; MG, model group; EG, exercise group; NG, Nrf2 inhibitor group; ENG, exercise plus Nrf2 inhibitor group.

Microscopic images further demonstrated that ARF1 and ATP6V0A1 were densely and abundantly distributed in cells of the DC group, whereas their distribution was weak and sparse in the MG group (Figure 5A,C,F,H). These results indicate that the expression levels of ARF1 and ATP6V0A1 in skeletal muscle lysosomes were significantly reduced in the MG group. ELISA results (Figure 6A) showed that malondialdehyde (MDA) (G0109W48, Grace) levels were significantly elevated in the MG group when compared with the DC group (*p* < 0.05). Western blot analysis (Figure 6B) revealed that the protein expression levels of LDH-A were significantly increased in the MG group relative to the DC group (*p* < 0.01 or *p* < 0.05).

### 3.3. Exercise Is Associated with Changes in ARF1-Related Autophagy–Lysosome Markers in Skeletal Muscle of Long-Term HFD-Fed Mice

As shown in Figure 1B, during weeks 1–6 of intervention, body weights in the MG group were higher than those in the DC group (*p* < 0.05). Starting from the 5th week of intervention, the body weights in the EG group were significantly lower than those in the MG group (*p* < 0.01). The results showed that after 6 weeks of intervention, the EG group exhibited significantly decreased TC, TG, LDL-C, FBG, FINS and HOMA-IR levels compared with the MG group (*p* < 0.01). TC, TG, LDL-C, FBG, FINS and HOMA-IR levels were markedly reduced in the ENG group (*p* < 0.01) (Figure 2), suggesting that exercise significantly improves the abnormalities of lipid indicators induced by long-term HFD. H&E staining of skeletal muscle revealed that, compared with the MG group, the EG group showed narrower intercellular gaps between muscle fibers, fewer intracellular vacuoles, and a more intact cellular structure. Moreover, the proportion and area of muscle tissue significantly increased (*p* < 0.01), indicating that exercise can improve the morphology of skeletal muscle (Figure 3A–D). As shown in Figure 4, compared with the MG group, the mRNA expression levels of *Arf1*, *Atp6v0a1*, *Ldh-b* and *Ulk1* were significantly upregulated in the EG group (*p* < 0.05 or *p* < 0.01), while *Ldh-a* mRNA expression was significantly decreased (*p* < 0.05 or *p* < 0.01). Immunofluorescence results (Figure 5) demonstrated that, relative to the MG group, the EG group exhibited markedly increased expression levels and positive staining areas of ARF1 and ATP6V0A1 in skeletal muscle lysosomes, suggesting that aerobic exercise may elevate ARF1 and ATP6V0A1 expression in HFD mice. There was significantly enhanced colocalization between ARF1 and Lamp1 in the EG group relative to the MG group (as shown in Figure 5E,J). ELISA results (Figure 6A) showed that L-lactate dehydrogenase (L-LDH) (AKCO003M, Boxbio, Beijing, China) levels show a significant upward trend in the EG group compared with the MG group. The glutathione peroxidase (Gpx, G0204W48, Grace, Suzhou, China) and MDA content was significantly reduced in the ENG group when compared with the MG group (*p* < 0.05). Western blot analysis revealed that, compared with the MG group, the EG group displayed significantly elevated protein expression of Nrf2, ULK1, and LDH-B (*p* < 0.05 or *p* < 0.01), while the expression of LDH-A was significantly decreased (*p* < 0.05). Exercise significantly increased the levels of Nrf2 and LDH-B in skeletal muscle, enhanced the expression of autophagy-related proteins including ULK1, and downregulated LDH-A expression. Collectively, these findings indicate that exercise is associated with morphological improvements in skeletal muscle from long-term HFD-fed mice, likely linked to changes in ARF1-related autophagy–lysosome markers.

### 3.4. Associations Between ARF1-Related Autophagy–Lysosome Markers and Nrf2 Signaling

ARF1 participates in the regulation of ALR and mitophagy, ensuring the continuous and efficient progression of autophagic flux [10]. Nrf2 is a core transcription factor responsible for cellular defense. Under oxidative stress, Nrf2 translocates into the nucleus and initiates the expression of a series of cytoprotective genes to enhance antioxidant and anti-inflammatory capacities [28]. To explore associations between Nrf2 signaling and ARF1-linked autophagy–lysosome markers, an Nrf2 inhibitor was applied, and changes in ARF1 localization relative to lysosomes were examined. As shown in Figure 3, muscle cells in the NG group under high-fat conditions were loosely arranged, with irregular tissue morphology and numerous intracellular vacuoles; the area of the muscle tissue had decreased. In the ENG group, muscle cell arrangement became more regular and intracellular vacuoles were reduced, and the muscle-tissue area increased, suggesting that exercise improves skeletal muscle morphology. As shown in Figure 4, compared with the EG group, the NG group exhibited significantly decreased mRNA expression of *Arf1*, *Atp6v0a1*, *Ldh-b* and *Ulk1* (*p* < 0.05 or *p* < 0.01) and markedly increased mRNA expression of *Ldh-a* (*p* < 0.01). The mRNA levels of *Ulk1* were significantly reduced in the ENG group compared with the NG group (*p* < 0.05 or *p* < 0.01). The ENG group showed significantly upregulated mRNA expression of *Arf1* when compared with the NG group (*p* < 0.01). Figure 5 shows that ARF1 expression was decreased and ATP6V0A1 expression was slightly increased in the NG group; both ARF1 and ATP6V0A1 levels were moderately increased in the ENG group. ELISA results showed that L-LDH levels were significantly decreased in the NG group compared with the EG group (*p* < 0.05). Western blot analysis revealed that the protein expression of Nrf2 was significantly reduced in the NG group relative to the EG group (*p* < 0.01 or *p* < 0.05). Compared with the NG group, the ENG group exhibited significantly decreased protein expression of Nrf2, ATP6V0A1, LDH-A, LDH-B, and ULK1 (*p* < 0.01 or *p* < 0.05) (Figure 6).

## 4. Discussion

Lysosomes have long been regarded as cellular waste-processing compartments responsible for degrading macromolecules and impaired organelles. Nevertheless, their biological roles extend far beyond degradation. Growing experimental evidence indicates lysosomes serve as central metabolic hubs, participating in the sensing and transmission of nutrient signals [29]. Under different nutritional conditions, the functional switch between mTORC1-mediated lipogenic anabolic and catabolic signaling pathways expands lysosome function from a degradation hub to a nutrient-sensing platform. Lysosomes sequentially remodel their lysosomal morphology and function, and activate mTORC1 to coordinate lipid anabolic and catabolic metabolism [30]. The mammalian target of mTOR represents one of the best-characterized signaling cascades governing metabolite sensing and signal transduction. Amino acid-induced activation of the mTORC1 complex relies on two distinct pathways, Rag GTPase and ARF1, which mediate lysosomal translocation of mTORC1 via v-ATPase. Moreover, studies using HFD-fed mouse models have demonstrated that mTOR signaling is upregulated owing to the recruitment of mTORC1 to lysosomes via ARF1 [9]. HFD exposure elicits multiple adverse systemic effects, including reduced skeletal muscle mass, compromised insulin sensitivity, and impaired exercise capacity. These detrimental phenotypes are mainly driven by HFD-induced lipotoxicity, mitochondrial dysfunction, and oxidative stress. Notably, defective lysosomal remodeling during autophagy is recognized as one of the core underlying mechanisms [31]. Our findings indicate that long-term HFD induces disorders in glucose and lipid metabolism, increases insulin resistance, and triggers morphological alterations in skeletal muscle accompanied by changes in ARF1. These findings further validate the pathological role of HFD in promoting skeletal muscle injury.

As a pivotal hub in the regulation of cellular metabolism, the mTORC1 pathway exerts an important role in multiple diseases, including cancer and diabetes [32]. Studies have uncovered a novel molecular mechanism whereby ARF1 transmits nutrient-sensing signals upstream of the mTORC1 pathway. Specifically, cellular glucose metabolism modulates ARF1 lactylation, which may govern ARF1-associated functions. Such functions encompass ARF1-mediated ALR [10] and intercellular mitochondrial transfer [11]. RT-qPCR and immunohistochemistry results demonstrated that the mRNA expression levels of *Arf1*, *Atp6v0a1* and *Ulk1* were significantly decreased in the skeletal muscle of HFD-fed mice. In contrast, Western blot analysis showed no significant differences in the protein abundance of ARF1, ATP6V0A1, and ULK1 relative to the control group. The discrepancy between mRNA and protein levels may partly stem from multilayered proteostatic buffering under HFD stress. For instance, transcriptional changes may be counterbalanced by translational repression, and enhanced degradation of newly synthesized proteins could offset alterations in transcript abundance, potentially contributing to the maintenance of steady-state protein levels [33]. Alternatively, discordant mRNA expression may arise from transcription–translation decoupling mediated by stress granule sequestration or miRNA-dependent silencing [34]. Static Western blot measurements fail to capture these dynamic protein turnover processes, highlighting the need for future pulse-chase experiments. In addition, prior studies have reported aberrant upregulation of LDH-A alongside suppressed LDH-B expression in skeletal muscle under chronic metabolic disease conditions [35]. This dysregulated expression profile shifts overall L-LDH activity toward anaerobic glycolysis, consequently leading to intracellular lactate accumulation, increased reactive oxygen species (ROS) production, and the induction of lipid peroxidation, accompanied by marked elevations in MDA levels [36]. Such alterations trigger compensatory upregulation of GPx transcription via the Nrf2/ARE pathway [37]. Our results demonstrated increased levels of LDH-A, GPx, and MDA, as well as reduced LDH-B expression, in the skeletal muscle of HFD-fed mice. As a master regulator of antioxidant stress, Nrf2 engages in complex, bidirectional crosstalk with the mTORC1 pathway.

This regulatory relationship comprises two layers: mTORC1-mediated modulation of Nrf2 and the feedback regulation of mTORC1 by Nrf2 [38,39]. Notably, Nrf2 is relatively sensitive to mTOR activity. For example, studies in renal cancer cells have demonstrated that mTOR suppresses Nrf2-dependent antioxidant responses, suggesting a regulatory function of mTORC1 [40,41]. In the setting of selective autophagy, the mTORC1 pathway has been reported to promote the activation of Nrf2 [42,43]. Conversely, oxidative stress can upregulate Nrf2 in an mTORC1-independent manner [44,45]. Notably, mTORC1 activation correlates with increased ROS production, and excessive ROS accumulation may indirectly inhibit the Nrf2 signaling pathway [46,47]. In turn, Nrf2 can positively regulate autophagy by modulating the expression of autophagy-related genes such as p62 [42,43]. Taken together, these findings reveal correlated alterations in Nrf2 signaling and autophagy-related markers, accompanied by concurrent changes in ARF1. Our previous work has demonstrated that exercise serves as an effective intervention to activate Nrf2 [34]. As a form of controllable oxidative stress, exercise can trigger the Nrf2 signaling pathway, boost the antioxidant capacity of skeletal muscle, and alleviate HFD-induced oxidative damage [34]. Treatment with luteolin to inhibit Nrf2 signaling diminished the protective effects of exercise on skeletal muscle injury under prolonged HFD conditions. Additionally, autophagy underpins the metabolic benefits conferred by exercise. Specifically, exercise activates autophagy through multiple signaling molecules including AMPK [48], CaMK, and SIRT1. These mediators promote the clearance of lipid droplets and damaged mitochondria, thereby improving skeletal muscle function. Through metabolic reprogramming, exercise increases lactate production, and the lactylation modification of proteins such as mTOR suppresses mTORC1 activity and consequently initiates autophagy [18]. In the present study, we found that exercise upregulated the mRNA levels of *Arf1* and *Atp6v0a1*, as well as both mRNA and protein expression of LDH-B and ULK1. Moreover, exercise increased the colocalization of ARF1 and ATP6V0A1 with Lamp1, while decreasing LDH-A expression. Collectively, these findings indicate that exercise is linked to attenuated oxidative stress and altered autophagy-related markers in skeletal muscle.

## 5. Limitations and Prospects

The main limitations of the present study are as follows: First, we did not thoroughly investigate the specific transcriptional binding sites through which Nrf2 regulates ARF1. Second, regarding the experimental group design, we lacked a normal diet + luteolin group and a normal diet + exercise group. Accordingly, we cannot clearly distinguish the baseline effects of luteolin monotherapy and exercise alone on the measured indicators under healthy physiological conditions. Third, luteolin was used as a pharmacological Nrf2 modulator, yet it lacks strict selectivity; its inherent anti-inflammatory and antioxidant activities may interfere with the results, and genetic manipulation of Nrf2 is needed to confirm its specific causal role. Fourth, this study only revealed a correlative relationship between Nrf2 and ARF1, without verifying their causal regulatory direction and the direct transcriptional binding sites of Nrf2 on ARF1. In addition, direct ARF1 lactylation modification was not detected, and relevant verification experiments are still required. Fifth, although the expression and colocalization of key autophagic proteins were examined, direct autophagic flux and lysosomal reformation assays were not conducted, so the conclusions about autophagic activity remain preliminary. Finally, skeletal muscle functional indicators including contractile function, grip strength and fatigue resistance were not measured, and the observed molecular and structural changes cannot fully confirm functional deficits.

## 6. Conclusions

Long-term HFD may induce morphological alterations in skeletal muscle, accompanied by changes in ARF1-associated autophagy–lysosome markers and elevated oxidative stress. Conversely, moderate-intensity exercise is associated with shifts in markers related to autophagosome–lysosome interactions and ARF1 localization, alongside reduced lipid droplet accumulation in HFD-exposed skeletal muscle. In summary, exercise is associated with skeletal muscle autophagy–lysosome-related markers and Nrf2/ARF1 signaling in HFD mice.

## Figures and Tables

**Table 1:** Sequence of gene expression primers determined by real-time PCR.

Target Genes		Genetic Sequences
*Arf1*	Forward primer 5’-3’	TCAGCTTCACCGTGTGGGATGT
	Reverse primer 5’-3’	CCTCGTTCACACGCTCTCTGTC
*Atp6v0a1*	Forward primer 5’-3’	CTGTTATCCTCGGCATCATCCAC
	Reverse primer 5’-3’	CAGGTAGCCAAACAACGAGGAC
*Ldh-a*	Forward primer 5’-3’	ACGCAGACAAGGAGCAGTGGAA
	Reverse primer 5’-3’	ATGCTCTCAGCCAAGTCTGCCA
*Ldh-b*	Forward primer 5’-3’	CCTCAGATCGTCAAGTACAGCC
	Reverse primer 5’-3’	ATCCGCTTCCAATCACACGGTG
*Ulk1*	Forward primer 5’-3’	GCAGCAAAGACTCCTGTGACAC
	Reverse primer 5’-3’	CCACTACACAGCAGGCTATCAG
*β-actin*	Forward primer 5’-3’	GCTCTGGCTCCTAGCACCAT
	Reverse primer 5’-3’	GCCACCGATCCACACAGAGT

## Data Availability

The original contributions presented in this study are included in the article/Supplementary Materials. Further inquiries can be directed to the corresponding authors.

## Supplementary Materials

The following supporting information can be downloaded at https://www.mdpi.com/article/10.3390/nu18203329/s1. File S1: The changes in body weight of different groups after the intervention.
